# Developing a Novel, Green, and Efficient Synthesis Method for Polycarboxylate Superplasticizers Through Mechanochemical Internal Mixing Polymerization

**DOI:** 10.3390/polym17081017

**Published:** 2025-04-09

**Authors:** Qianqian Chen, Xiaomiao Li, Lisha Pan, Chang Lin

**Affiliations:** School of Chemistry and Chemical Engineering, Hainan University, Haikou 570228, China; 22210817000003@hainanu.edu.cn (Q.C.); lixm6424@163.com (X.L.); clin@hainanu.edu.cn (C.L.)

**Keywords:** mechanochemical internal mixing polymerization, polycarboxylate superplasticizer, energy consumption, fluidity

## Abstract

Polycarboxylate superplasticizers (PCEs) are the most important polymer admixtures in cement and concrete. Developing novel, green, and efficient synthesis methods is essential for lowering energy consumption. Here, a mechanochemical internal mixing polymerization was used to synthesize high-concentration PCEs (INPCEs) for the first time. The optimum reaction temperature, reaction rotating speed, and reaction time were determined using the orthogonal method. The optimum acid–ether ratio (i.e., the molar ratio of acrylic acid (AA) to isopentenyl polyoxyethylene ether (TPEG)) and concentrations of ammonium persulfate (APS) and sodium methacrylate sulfonate (MAS) were also determined. Finally, the molecular structures of the INPCEs were characterized using Fourier transform infrared spectroscopy (FT-IR) and gel permeation chromatography (GPC), and their performance and energy consumption were compared with PCE synthesized via an aqueous solution polymerization (TPCE). The results showed that the optimum reaction temperature, rotating speed, and time were 60 °C, 70 R/min, and 60 min, respectively. In addition, the acid–ether ratio, the concentrations of MAS and APS, and the polymerization method affected the molecular weight and PDI of INPCEs but did not alter the functional groups. At an AA:TPEG:MAS molar of 3.0:1:0.12 and an APS concentration of 1 wt% (relative to TPEG), the initial fluidity of cement paste with INPCE was 312.5 mm at an INPCE dosage of 0.20 wt% and a water–cement ratio of 0.35. Further, the concentrations of the INPCEs were >99.00 wt%, which is much higher than the TPCE concentration of 39.73 wt%, and the dispersion and dispersion retention of INPCE was almost as good as that of TPCE while requiring much less energy for synthesis. These findings can contribute to the reduction in energy consumption in the concrete industry.

## 1. Introduction

Polycarboxylate superplasticizers (PCEs) are the most commonly used admixtures in concrete due to their extensive research and application [1,2,3]. PCEs offer significant advantages such as good adaptability to concrete and improved concrete workability [2,4]. Meanwhile, due to their unique comb structure, they have good designability in molecular structure and are environmentally friendly to synthesize [1,5,6].

**Figure 1:**
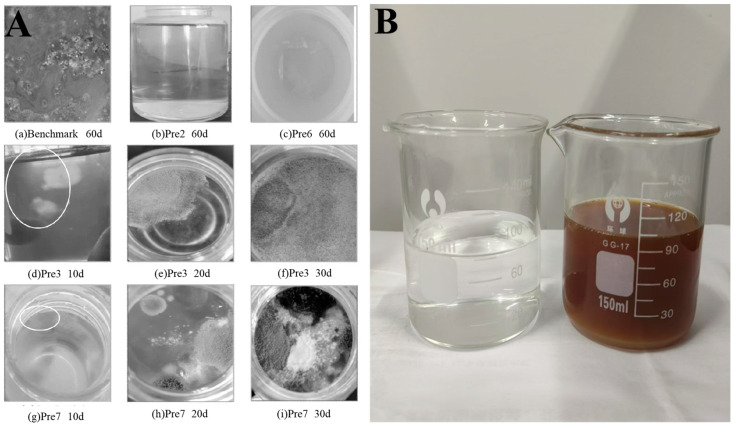
(**A**) Changes in the appearance of stored PCE samples over time [15], (**B**) PCE samples before (left) and after (right) mildew formation.

At present, PCEs are mainly synthesized via aqueous solution polymerization. However, the reaction time of this method is over 4 h [6,7,8] and it typically yields low product concentrations of only 10−50 wt% [9,10,11,12], resulting in increased transportation and storage costs [13,14]. In addition, due to the addition of carbohydrates as a retardant, microorganisms in PCE solutions tend to multiply and metabolize in large quantities during the hot summer months. This causes the liquid to turn black in color and give off a bad smell as well as to develop mildew on its surface, as illustrated in Figure 1 [15,16]. As a result, the water-reducing performance of PCEs is severely impacted [16,17,18] and their aqueous solutions cannot be directly applied to dry mortar, high-strength grouting, thermal insulation mortar, plastering mortar, or ceramic bonding mortar [12,19].

**Figure 2:**
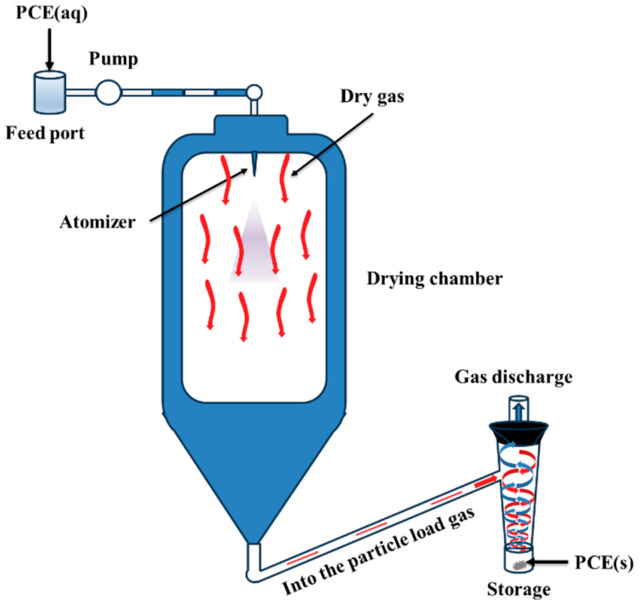
Process flow diagram of spray drying.

To overcome these issues, research efforts to develop more efficient synthesis methods capable of directly obtaining high PCE concentrations are underway. At present, high-concentration PCEs are commonly obtained through spray drying, as illustrated in Figure 2 [20]. Pang et al. [21] prepared powdered polycarboxylate superplasticizers (PCC-h) with high dispersion using a high-speed centrifugal spray dryer with an inlet air temperature of 300 °C and a fixed outlet temperature of 90 °C. Wang et al. [22,23] synthesized a solid PCE (SPC) using a solvent precipitation method, which involved adding an organic solvent to precipitate the SPC from the solution before separating and purifying it. The appearance and molecular structure of the SPC product indicated the superior storage stability of SPCs. Zhu [24] prepared solid PCE using an adsorbent made from mineral dopants and organic resin to adsorb liquid PCE. Sun et al. [25] used a ball milling method to synthesize high-concentration PCEs (MPCEs), successfully obtaining a much higher concentration (>90 wt%) compared with the aqueous solution polymerization product. In addition, the fluidity of cement paste with MPCE synthesized using a ball milling time of 30 min was found to be over 260 mm at a dosage of 0.30 wt% and a water–cement ratio of 0.29. Zhang et al. [26] described a highly efficient eco-friendly microwave induction-based PCE synthesis method. Their results showed the green advances of PCE preparation by microwave induction in the preparation time and conversion rate, also providing good dispersion in fresh cement paste at a lower dosage. Nevertheless, the current preparation methods suffer from shortcomings such as the use of organic solvents, difficulties in the regeneration and recycling of adsorbents, and high costs [13,14]. By contrast, the internal mixing method offers the advantages of low energy consumption, high production efficiency, flexible operation, and high production output while using simple and easy-to-maintain equipment [27,28,29].

In this study, high-concentration PCEs (INPCEs) were synthesized via a mechanochemical internal mixing polymerization using ammonium persulfate (APS) as an initiator, sodium methacrylate sulfonate (MAS) as a chain transfer agent, and isopentenyl polyoxyethylene ether (TPEG) and acrylic acid (AA) as reactive monomers. The optimum reaction temperature, rotating speed, and time were determined using the orthogonal method. In addition, the effects of varying the acid–ether ratio and the concentrations of APS and MAS on the initial fluidity of cement paste were investigated, and Fourier transform infrared spectroscopy (FT-IR) and gel permeation chromatography (GPC) were used to characterize the molecular structures of the INPCEs. Finally, the effects of INPCEs on the concentration, initial fluidity, and loss of fluidity of cement pastes were compared with that of PCE-obtained aqueous solution polymerization (TPCE). The energy consumption of the two polymerization methods was also compared.

## 2. Materials and Experimental

### 2.1. Materials

Isopentenyl polyoxyethylene ether (TPEG), with an average molecular weight (*M*_w_) of 2400, was supplied by Nantong Hengchuang Chemical Co., Ltd. (Nantong, China). Acrylic acid (AA), sodium methacrylate sulfonate (MAS), and ammonium persulfate (APS) were obtained from Shanghai McLean Biochemistry Technology Co., Ltd. (Shanghai, China). Potassium bromide (KBr), was obtained from Xilong Science Co., Ltd. (Shantou, China). Sodium hydroxide (NaOH), was obtained from Xilong Chemical Co., Ltd. (Shantou, China). Sodium nitrate (NaNO_3_), was obtained from Guangdong Guanghua Science and Technology Co., Ltd. (Guangdong, China). Hydroquinone (HQ) was obtained from Xiya Reagent, Shandong, China. Apart from TPEG, which was industrial grade, all other reagents were analytical reagents (AR). Ultrapure water (H_2_O) was prepared in the laboratory using the GWA-UN4-F20 ultrapure water dispenser (Beijing Puxi General Instrument Co., Ltd., Beijing, China) Experiments measuring the fluidity and fluidity loss of cement paste were conducted using the benchmark cement of the national standard GB8076-2008 [30] (Table 1).

### 2.2. Synthesis of PCEs

#### 2.2.1. Orthogonal Test

**Figure 3:**
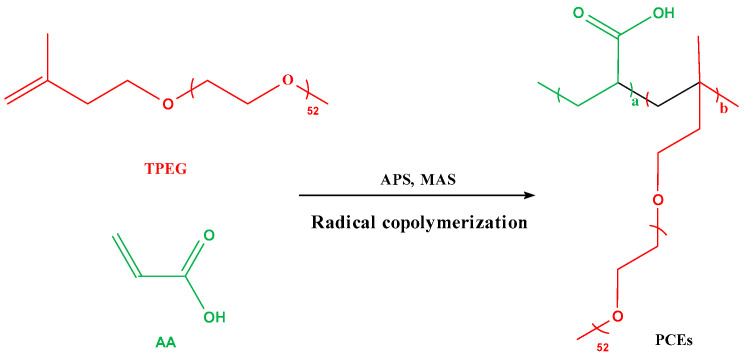
Polymerization reaction equation for PCEs.

**Figure 4:**
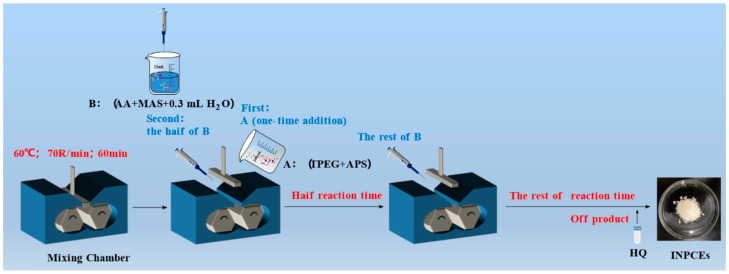
Polymerization scheme for INPCEs.

The synthesis of INPCEs was carried out by using an XSS-300 torque rheometer (Shanghai Kechuang Rubber & Plastic Machinery Equipment Co., Ltd., Shanghai, China). The chamber was first set to the desired reaction temperature and rotational speed before adding solid mixture A (TPEG + APS) and allowing it to fully melt. Next, half of the liquid mixture B (AA + MAS + H_2_O) was added to the chamber, and the rest was added once half of the reaction time had elapsed. To terminate the reaction, HQ (0.03 wt% of the total amount of TPEG) was added, the system was brought down to room temperature, and the pH was adjusted to ~7 using a 30 wt% NaOH solution. Figure 3 and Figure 4 show the polymerization reaction equation and the polymerization scheme, respectively.

Three factors, namely the reaction temperature, rotating speed, and time, were used for the orthogonal test, and an L_9_ (3^4^) orthogonal array was designed to determine the optimum values of these factors by testing the initial fluidity of cement paste. Table 2 shows the levels of the factors in the orthogonal test.

#### 2.2.2. Synthesis of INPCEs

Table 3 shows the reactant ratios and reaction parameters used to synthesize the different INPCEs. In total, 3 sets of INPCEs were synthesized, labeled as INPCEs A, B, and C, respectively, with five samples in each set. In set A the acid–ether ratio was varied, while set B consisted of INPCEs synthesized with different masses of initiator, and set C included INPCEs synthesized with different molar masses of the chain transfer agent. Finally, the reaction was terminated using the same procedure as described in Section 2.2.1.

### 2.3. Synthesis of TPCE

**Figure 5:**
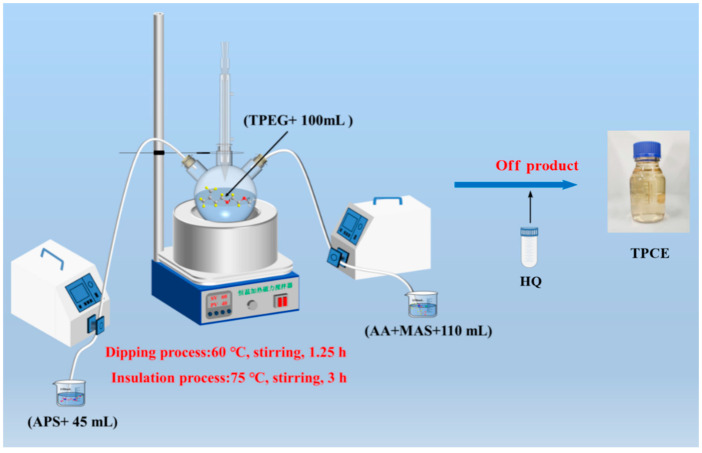
Polymerization scheme for TPCE.

To synthesize the TPCE, TPEG was dissolved in water in a three-necked flask equipped with a condenser tube under continuous stirring at 60 °C. The prepared initiator solution A (APS + H_2_O) and monomer mixture solution B (AA + MAS + H_2_O) were then dropped into the flask, taking ~75 min and 60−70 min to finish dropping, respectively. After dropping, the temperature was raised to 75 °C and held for 3 h [31]. Finally, the reaction was terminated using the same procedure as described in Section 2.2.1. The relevant reactant ratios and reaction parameters are listed in Table 4 and the polymerization scheme is illustrated in Figure 5.

### 2.4. Purification of PCEs

The synthesized PCEs were continuously dialyzed for 3 days through an 8000 Da dialysis bag. The liquid retained in the dialysis bag was removed and placed in a SCIENTZ-18 vacuum freeze dryer (Ningbo Xinzhi Bio-technology Co., Ltd., Ningbo, China) to obtain the purified sample. This method can remove unreacted monomers and salts in PCEs. The purified samples were used for FT-IR testing.

### 2.5. Characterization of PCEs

#### 2.5.1. Fourier Transform Infrared Spectroscopy (FT-IR)

Vacuum freeze-dried PCE powder and KBr were mixed in a mass ratio of 1:100 and ground to powder form under an infrared baking lamp before being pressed into thin tablets using a tablet press. The FT-IR measurements were carried out using an FTIR-650 (Tianjin Gangdong Science and Technology Development Co., Ltd., Tianjin, China).

#### 2.5.2. Gel Permeation Chromatography (GPC)

GPC measurements were carried out using a Waters 1515 gel chromatograph (Waters Corporation, Milford, MA, USA) equipped with a Waters 2414 oscillometric refractive detector. The column temperature was set to 40 °C with a flow rate of 0.6 mL/min. In these tests, 1.0 wt% PCE solution was filtered through a 0.45 μm filter, and 0.1 mol/L NaNO_3_ aqueous solution was used as the mobile phase. Different relative molecular masses of dextran were chosen as the standards, and the relative molecular masses of the polymers were obtained from the peak times of GPC spectra.

### 2.6. Performance Test of PCEs

#### 2.6.1. Concentration Test

An MB45 moisture meter (Ohaus, Parsippany, NJ, USA) was used to measure the concentration of PCEs. In these tests, the sample disk was first placed in the moisture meter and the mass was zeroed. A PCE sample greater than 0.5 g was then placed in the sample disk and its concentration was recorded until it reached a constant weight.

#### 2.6.2. Initial Fluidity

The initial fluidity of cement paste samples was measured according to the national standard GB8077-2012 [32]. (“Test Method for Homogeneity of Concrete Admixtures”) using an NJ-160 cement net slurry mixer (Wuxi Jiangong Experimental Ltd., Wuxi, China). The INPCEs in the orthogonal test (Table 2) were tested at a water–cement ratio of 0.35 and a PCE dosage of 0.15 wt%, while INPCEs and TPEG samples not in the orthogonal array were tested at a water–cement ratio of 0.35 and a PCE dosage of 0.20 wt%.

#### 2.6.3. Calculation of Fluidity Loss

After the initial fluidity tests, the cement paste was collected and poured back into the mixing pot, and the mouth of the pot was covered with a wet cloth. After standing at room temperature and natural humidity for a specified time, the mixing pot was added to the mixer under slow stirring for 2 min. Every 30 min until 120 min, the fluidity was measured; the measured fluidity is the fluidity over time. The percentage loss of fluidity of the cement paste (*X*) is calculated as follows:

(1) $$X=\frac{F_{0}-F_{t}}{F_{0}}\times 100\%$$

where *F*_0_ is the initial fluidity of cement paste. *F_t_* is the fluidity after a settling time t has elapsed.

### 2.7. Energy Consumption

The data indicators (Table 4) used for the energy consumption calculations came from the performance indicators (Table 5) in this study and the experimental data of various laboratory equipment. The processes involved in PCE fabrication from cradle to gate, including the extraction and processing of raw materials, the production of chemical precursors, and the production of the PCE polymer itself, were examined using a simplified life cycle assessment (LCA) approach [33,34,35].

For instance, let us compare the energy consumption of the mechanochemical internal mixing polymerization and aqueous solution polymerization required to synthesize a given mass of solid PCE. Since the difference in raw material usage between the two methods is not significant (Table 4), the energy consumption of the raw materials is assumed to be the same for each method. Table 5 lists the power ratings of the instruments involved in PCE synthesis. The energy consumption equations are as follows:

*E* = *E*_a_ + *E*_0_ (2)

(3) $$E_{a}=\sum_{i=1}^{n}P_{i}\times t_{i}$$

where *E* is the total energy consumption required to produce a mass of solid PCE (kW·h). *E*_a_ is the energy required to synthesize solid PCE. *E*_0_ is the energy consumption of raw materials. *P_i_* and *t_i_* are the power rating (kW) and usage time (h) of instrument *i*, and *n* is the number of instruments used for a particular method (*n* = 1 for INPCE and *n* = 5 for TPCE).

## 3. Results and Discussion

### 3.1. Orthogonal Test

Table 6 shows the orthogonal experimental results, whence it can be seen that the order in which the three factors affect the initial fluidity of cement paste with INPCE is reaction time > reaction temperature > reaction rotating speed. The values of these factors for which the optimal dispersibility was obtained were 60 °C (denoted as A1), 70 R/min (denoted as B2), and 60 min (denoted as C1), respectively. Therefore, the optimal combination is A1B2C1. The following tests were carried out using these process parameter values.

### 3.2. Structural Characterization

#### 3.2.1. FT-IR

**Figure 6:**
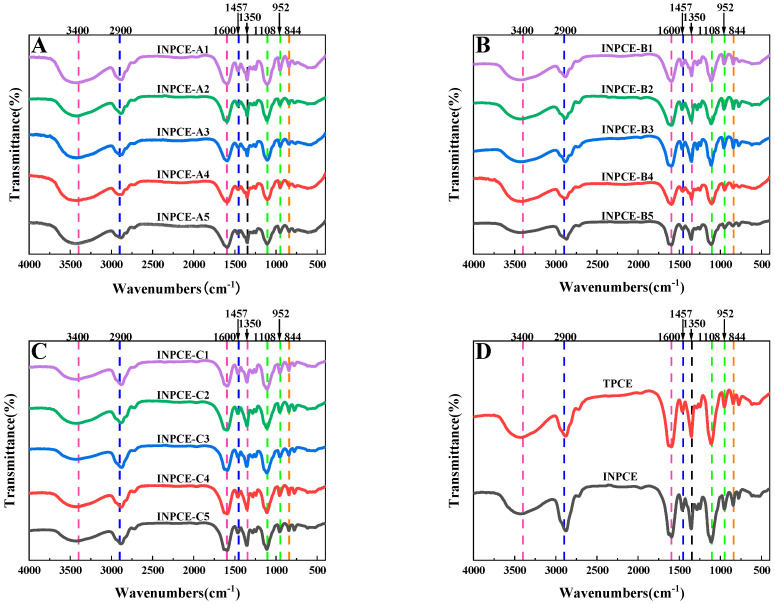
FTIR spectra of purified PCEs: (**A**) INPCE-A, (**B**) INPCE-B, (**C**) INPCE-C, and (**D**) INPCE and TPCE.

Figure 6 shows the FT-IR analysis results of the INPCE and TPCE samples. In all cases, the -OH and C=O stretching vibration peaks of the carboxyl group (-COOH) were observed at 3400 cm^−1^ and 1600 cm^−1^. The C-H stretching and bending vibration peaks of alkyl (-CH_2_-) were observed at 2900 cm^−1^ and 1457 cm^−1^, respectively. The C-H bending vibration of methyl (-CH_3_) was observed at 1350 cm^−1^. The C-O-C stretching and bending vibration peaks were observed at 1108 cm^−1^ and 952 cm^−1^, respectively. The -CH_2_CH_2_O- bending vibration peak was observed at 844 cm^−1^. These results are consistent with the literature [36,37]. Further, Figure 6A–C show that changing the acid–ether ratio and the concentrations of APS and MAS does not affect the functional groups of INPCEs. Meanwhile, Figure 6D shows that the choice of polymerization method does not affect the functional groups of PCEs, as both INPCE and TPCE had the same functional groups.

#### 3.2.2. GPC

**Figure 7:**
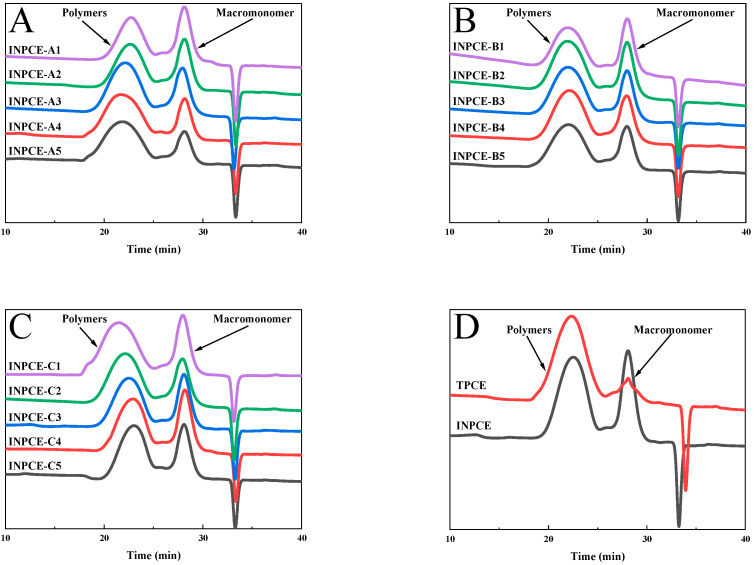
GPC spectra of PCEs: (**A**) INPCE-A, (**B**) INPCE-B, (**C**) INPCE-C, and (**D**) INPCE and TPCE.

**Figure 8:**
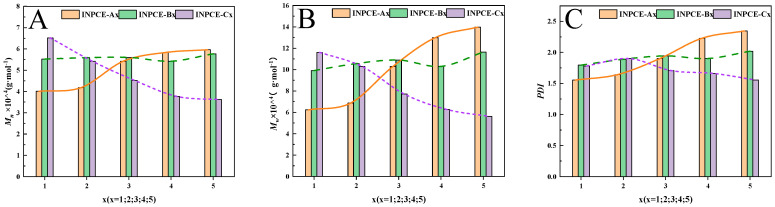
(**A**) *M*_n_, (**B**) *M*_w_, and (**C**) PDI of INPCEs.

Figure 7 shows the GPC spectra of the three sets of INPCEs as well as the TPCE, with the corresponding values of number average molecular weight (*M*_n_), *M*_w_, and polymer dispersity index (PDI) provided in Figure 8 and Table 7 and Table 8. As Table 8 shows, the *M*_w_ and PDI values of the INPCEs were in the range of 57,000–140,000 g·mol^−1^ and 1.55–2.35, respectively. Further, it is evident that *M*_w_ and PDI are most strongly influenced by the acid–ether ratio and the MAS concentrations and less so by the APS concentrations. As Table 7 shows, TPCE has higher *M*_w_ and PDI compared with INPCE. Overall, the FT-IR and GPC results show that the INPCEs and TPCE were successfully synthesized.

Figure 8 shows that the *M*_n_, *M*_w_, and PDI of INPCE-A samples increased with increasing acid–ether ratio, which may be due to the fact that AA is more reactive than TPEG [38]. As the acid–ether ratio increases, the more reactive AA monomer participates in the polymerization reaction, resulting in an increase in molecular weight and PDI [38]. The overall slight upward trends in the *M*_n_, *M*_w_, and PDI of INPCE-B samples with increasing APS concentration are attributed to an increase in the number of monomers initiating polymerization [25]. Meanwhile, the *M*_n_, *M*_w_, and PDI of INPCE-C samples decreased with increasing MAS concentration due to the increased probability of chain transfer reactions in the growing polymer chain [39].

### 3.3. Fluidity of Cement Paste with INPCEs

**Figure 9:**
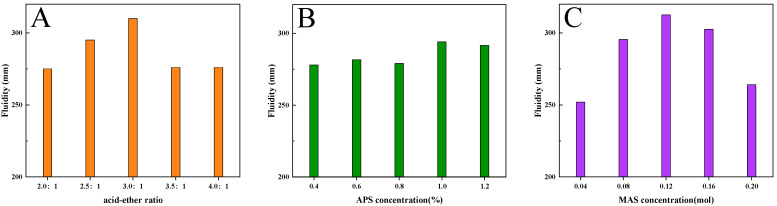
Effects of (**A**) acid–ether ratio, (**B**) APS concentration, and (**C**) MAS concentration on the initial fluidity of cement paste with INPCEs.

Figure 9 shows the effects of different reactants on the initial fluidity of cement paste with INPCEs. From Figure 9A, it can be seen that the initial fluidity of the cement paste with INPCEs shows a maximum value of 310 mm at an acid–ether ratio of 3.0:1. This may be because if the acid–ether ratio is too small, the side chain density is high, which facilitates entanglement between the side chains of INPCE molecules [40]. Simultaneously, there are fewer negatively charged adsorbent groups (-COOH) on the main chain, resulting in fewer INPCE molecules being adsorbed on the surface of the cement particles. This produces a weak static repulsion, thus weakening the initial fluidity of the cement paste [41,42,43]. Conversely, if the acid–ether ratio is too large, the higher amounts of negatively charged adsorbent groups on the main chain along with the lower density of the INPCE cause the side chain to curl up due to hydrogen bonding. As a result, part of the carboxyl group is wrapped up in the molecular chain, which reduces its contact area with the cement particles. Simultaneously, the spatial site resistance is weakened, which causes the initial fluidity of cement paste to decrease [41,42,43].

Figure 9B shows that as the APS concentration increases, the initial fluidity of cement paste shows a maximum value of 294 mm at 1 wt% APS (relative to TPEG). This is because when the amount of initiator is too low, the small number of primary radicals results in an incomplete copolymerization reaction, which lowers the initial fluidity of cement paste [44]. Conversely, if the initiator concentration is too high, the large number of primary radicals tends to induce the self-polymerization of small monomers, thereby reducing the degree of the copolymerization reaction, This results in a less-than-ideal molecular mass distribution of the synthesized product, which decreases the initial fluidity of the cement paste [38,45].

Figure 9C shows that the best fluidity of 312.5 mm was achieved at a molar ratio of *n*_AA_:*n*_TPEG_:*n*_MAS_ = 3.0:1:0.12. This is because, as a chain transfer agent, the concentration of MAS determines the length of the main chain of the INPCE molecules. When its concentration is small, the molecular weight of INPCE is large and its main chain is long, leading to the excessive adsorption of cement particles by individual molecules. This results in flocculation, which decreases the initial fluidity of cement paste [43,45]. Conversely, a high concentration of MAS shortens the main chain of INPCE, weakening the spatial site resistance of INPCE molecules while simultaneously reducing the charged adsorption groups and the adsorption capacity, causing the initial fluidity of cement paste to decrease [43,45].

### 3.4. Effect of Different Synthesis Methods on the Performance of PCEs

#### 3.4.1. Concentration

Table 9 shows that the concentration of the synthesized INPCEs were all >99.00 wt%, which is much higher than the corresponding value for TPCE of 39.73 wt%. This is mainly because the synthesis of INPCEs occurs via bulk polymerization, in which only trace amounts of solvent are added during the synthesis process. On the other hand, during the synthesis of TPCE, the monomer needs to be completely dissolved, which requires the addition of a large amount of solvent.

#### 3.4.2. Fluidity Loss

**Figure 10:**
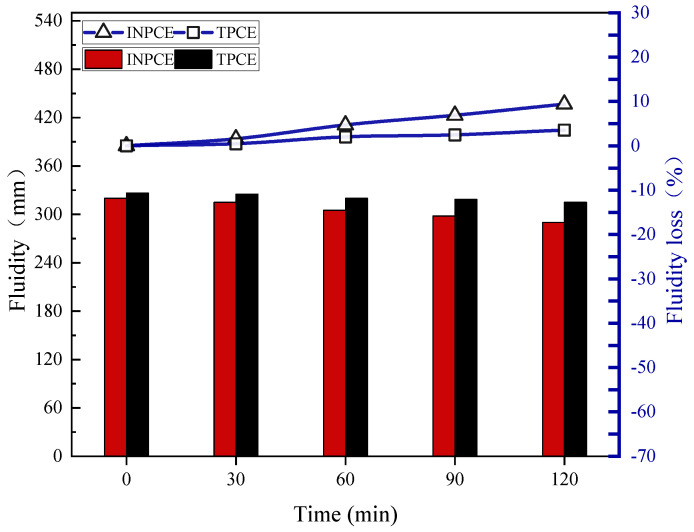
Effect of synthesis methods on the fluidity loss of cement paste.

Figure 10 shows the fluidity losses of cement pastes with different PCEs. The results show that after 120 min, the initial fluidity of cement pastes with INPCE and TPCE declined by 9.380% and 3.520%, respectively, from their initial values (from 320.0 mm to 290.0 mm and 326.5 mm to 315.0 mm, respectively). These results indicate that INPCE showed comparable dispersion and dispersion retention to TPCE.

#### 3.4.3. Energy Consumption

Table 10 shows the energy consumption of the two synthesis methods to produce a mass of solid PCEs, calculated using Equations (2) and (3). The results show that, based on the assumption that the raw material energy consumption is basically the same (i.e., *E*_0,INPCE_ ≈ *E*_0,TPCE_), the overall energy consumption for synthesizing INPCE is lower than that of TPCE (as *E*_a,INPCE_ = 5.00 kW·h while *E*_a,TPCE_ = 5.95 kW·h). This is due not only to the high energy efficiency and short synthesis time of mechanochemical synthesis methods but also to the fact that the synthesis of solid TPCE requires the removal of water, which increases energy consumption.

### 3.5. Reaction Mechanism and Prospects

**Figure 11:**
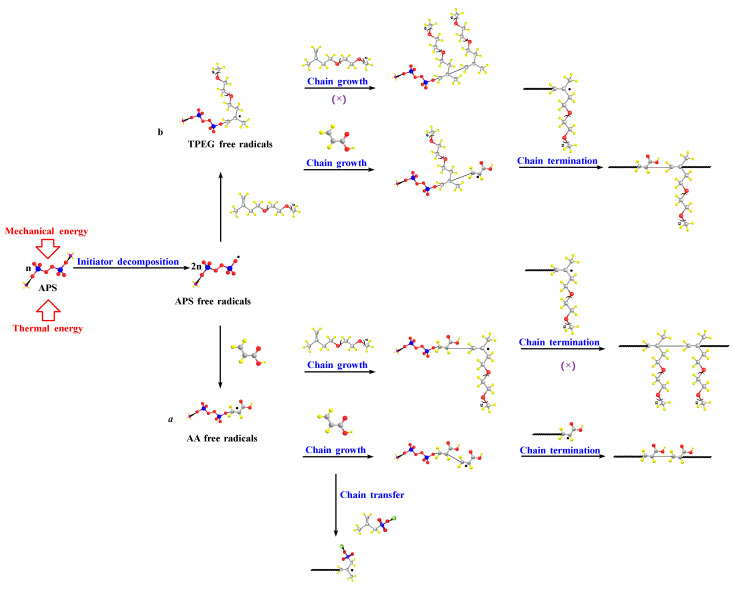
Polymerization mechanism of INPCE.

From the experimental results, it can be inferred that the polymerization mechanism of INPCE is free radical bulk polymerization, as illustrated in Figure 11. The polymerization process includes initiation, growth, transfer, and termination of the polymer chain. In the initiation step, APS generates primary free radicals under the dual action of thermal and mechanical energy, which form monomer radicals from the two monomers, AA and TPEG. In the growth phase, the monomer free radical opens a *π* bond in the monomer and adds on to it, forming a new radical. The activity of this new radical does not decay and it continues to add to the monomer chain to form an ever larger chain of radicals. Simultaneously, chain transfer occurs through the active center of the radical being transferred to other substances, thereby forming new radicals to continue chain growth. Finally, when the free radicals are too active to interact with each other, the reaction terminates, forming a macromolecule.

Theoretically, there are two types of chain initiation, four types of chain growth, and three types of chain termination in binary copolymerization systems [42]. However, combined with existing studies, it was shown that in the TPEG-AA binary copolymerization system, due to the lower activity of TPEG, there may be two chain initiations, three chain growths, and two chain terminations, as shown in Figure 11.

Free radical bulk polymerization is affected by several factors. For instance, when if the reaction temperature, the rotating speed, and the time are too high, both the decomposition efficiency of APS and the number of effective collisions between molecules increase. These conditions are conducive to implosion and the occurrence of side reactions, which leads to a reduction in the effective composition. By contrast, if APS cannot be fully decomposed effectively, the effective collision rate of molecules is low, and the reaction cannot fully occur, resulting in a lower effective composition. On the other hand, if the acid–ether ratio and the concentrations of the initiator and chain transfer agent are too high, the number of active small AA monomers in the system increases, as do the primary free radicals generated by APS. Under these conditions, small monomer self-polymerization readily occurs, and the degree of copolymerization reaction decreases. In addition, increasing the MAS concentration leads to an increase in chain transfer, which decreases the effective composition. By contrast, a higher content of TPEG in the system and a lower number of primary radicals result in an incomplete copolymerization reaction, while a lower content of MAS makes the molecular weight too large. Together, these factors also resulted in a decrease in the effective composition.

The synthesis method of INPCE is simple and efficient with low energy consumption and therefore leads to lower carbon emissions. With its high concentration and excellent performance, INPCE appears to be a promising candidate for a wide variety of industrial applications, pending further kinetic studies.

## 4. Conclusions

In this study, an efficient green mechanochemical internal mixing polymerization method was used to synthesize high-concentration INPCEs, and their performance and energy consumption were compared with TPCE. The main conclusions are as follows:

1. Varying the acid–ether ratio as well as the concentrations of MAS and APS affects the molecular weight and PDI of INPCEs. These properties are also dependent on the polymerization method used. However, the functional groups are the same regardless of the polymerization method.
2. The optimal process parameters were found to be a reaction temperature of 60 °C, a reaction rotating speed of 70 R/min, a reaction time of 60 min, *n*_AA_:*n*_TPEG_:*n*_MAS_ = 3.0:1:0.12, and an APS concentration of 1 wt% relative to TPEG.
3. The concentrations of all INPCEs were >99.00 wt%, which is much higher than that of TPCE. Further, INPCE showed similar excellent dispersion and dispersion retention properties as TPCE while requiring less energy to synthesize in the laboratory.

## Figures and Tables

**Table 1:** Chemical composition of the cement used in the experiments.

	Chemical Composition									
Composition	SiO_2_	Al_2_O_3_	Fe_2_O_3_	CaO	MgO	SO_3_	Na_2_Oeq	f−CaO	Loss	Cl^−^
Cement (wt%)	20.94	4.31	3.28	63.46	2.76	2.23	0.56	0.80	2.31	0.036

**Table 2:** Orthogonal experimental factors and levels design table.

Levels	Factors			
	A-Reaction Temperature (°C)	B-Reaction Rotating Speed (R/min)	C-Reaction Time (min)	D-Blank Column *
1	60	65	60	1
2	65	70	90	2
3	70	75	120	3

*: D is a blank group with no real intention.

**Table 3:** Reactant ratios and reaction parameters of synthesized INPCEs.

Samples		*n*_AA_ (mol)	*n*_TPEG_ (mol)	*n*_MAS_ (mol)	*m*_APS_ (g)	*V*_H2O_ (mL)	Reaction Temperature *T* (°C)	Reaction Rotating Speed (R/min)	Reaction Time (min)
INPCE-A	INPCE-A1	0.1750	0.0875	0.0070	2.10	1.5	60	70	60
	INPCE-A2	0.2188	0.0875	0.0070	2.10	1.5	60	70	60
	INPCE-A3	0.2625	0.0875	0.0070	2.10	1.5	60	70	60
	INPCE-A4	0.3063	0.0875	0.0070	2.10	1.5	60	70	60
	INPCE-A5	0.3500	0.0875	0.0070	2.10	1.5	60	70	60
INPCE-B	INPCE-B1	0.2625	0.0875	0.0070	0.84	1.5	60	70	60
	INPCE-B2	0.2625	0.0875	0.0070	1.26	1.5	60	70	60
	INPCE-B3	0.2625	0.0875	0.0070	1.68	1.5	60	70	60
	INPCE-B4	0.2625	0.0875	0.0070	2.10	1.5	60	70	60
	INPCE-B5	0.2625	0.0875	0.0070	2.52	1.5	60	70	60
INPCE-C	INPCE-C1	0.2625	0.0875	0.0035	2.10	1.5	60	70	60
	INPCE-C2	0.2625	0.0875	0.0070	2.10	1.5	60	70	60
	INPCE-C3	0.2625	0.0875	0.0105	2.10	1.5	60	70	60
	INPCE-C4	0.2625	0.0875	0.0140	2.10	1.5	60	70	60
	INPCE-C5	0.2625	0.0875	0.0175	2.10	1.5	60	70	60

**Table 4:** Reactant ratios and reaction parameters of PCEs.

Samples	*n*_AA_ (mol)	*n*_TPEG_ (mol)	*n*_MAS_ (mol)	*m*_APS_ (g)	*V*_H2O_ (mL)	Reaction Parameters
INPCE	0.2625	0.0875	0.0105	2.10	1.5	The reaction temperature was 60 °C, the reaction rotating speed was 70 R/min, and the reaction time was 60 min.
TPCE	0.2625	0.0875	0.0105	2.10	357.0	The reaction temperature was 60−75 °C, the stirring speed was 1600 R/min, and the reaction time was 255 min.

**Table 5:** Instruments used for synthesizing solid PCEs along with their power ratings.

Samples	Instrument Number	Instruments Names	Power Rating (kW)		Usage Time (h)
INPCE	1	XSS-300 torque rheometer from Shanghai Kechuang Rubber & Plastic Machinery Equipment Co., Ltd. with a 300mL mixing chamber (Shanghai, China).	4.00		1.25
TPCE	1	DF-101s Collector Type Constant Temperature Heating Magnetic Stirrer from Gongyi Yuhua Instrument Manufacturing Co., Ltd. (Gongyi, China).	Motor power	0.03	0.33
	2		Heating power	0.50	4.83
	3	DX-204 Low-Temperature Circulator from Beijing Changliu Scientific Instrument Co., Ltd. (Beijing, China).	0.30		4.83
	4	BT100-1L Peristaltic Pump Drive from Baoding Langer Constant Flow Pump Co., Ltd. (Baoding, China).	0.05		2.25
	5	OPD-8 Spray Dryer of Shanghai Dachuan Yuan Drying Equipment Co., Ltd. * (Shanghai, China).	9.80		0.20

*: Rated capacity of 3 kg/h.

**Table 6:** Orthogonal experimental results and analysis.

Experimental Number	A (Reaction Temperature)	B (Reaction Rotating Speed)	C (Reaction Time)	D (Blank Column)	Fluidity (mm)
1	1	1	1	1	237.5
2	1	2	2	2	237.5
3	1	3	3	3	232.5
4	2	1	2	3	210.0
5	2	2	3	1	212.0
6	2	3	1	2	230.0
7	3	1	3	2	228.0
8	3	2	1	3	247.0
9	3	3	2	1	208.0
k1	235.8	225.1	238.2	219.2	
k2	217.3	232.1	218.5	231.8	
k3	227.7	223.5	224.2	229.8	
R	18.50	8.667	19.67	12.67	
Primary and secondary order	C > A > B				
Excellent level	A_1_	B_2_	C_1_		
Excellent combination	A_1_ B_2_ C_1_				

**Table 7:** Molar weights and PDI of PCEs.

Samples	*M*_n_ (g·mol^−1^)	*M*_w_ (g·mol^−1^)	PDI
INPCE	45,179	77,074	1.71
TPCE	46,513	99,322	2.14

**Table 8:** The molar weights and PDI of INPCEs.

Samples		*M*_n_ (g·mol^−1^)	*M*_w_ (g·mol^−1^)	PDI
INPCE-A	INPCE-A1	40,074	62,257	1.55
	INPCE-A2	41,845	68,699	1.64
	INPCE-A3	54,188	102,996	1.90
	INPCE-A4	58,314	129,939	2.23
	INPCE-A5	59,575	139,677	2.34
INPCE-B	INPCE-B1	55,210	99,104	1.80
	INPCE-B2	55,925	105,609	1.89
	INPCE-B3	56,015	108,856	1.94
	INPCE-B4	54,188	102,996	1.90
	INPCE-B5	57,681	116,409	2.02
INPCE-C	INPCE-C1	65,174	116,062	1.78
	INPCE-C2	54,188	102,996	1.90
	INPCE-C3	45,179	77,074	1.71
	INPCE-C4	37,741	62,701	1.66
	INPCE-C5	36,151	56,187	1.55

**Table 9:** Concentration of synthesized PCEs.

Samples	INPCE-A					INPCE-B					INPCE-C					TPEG
	A_1_	A_2_	A_3_	A_4_	A_5_	B_1_	B_2_	B_3_	B_4_	B_5_	C_1_	C_2_	C_3_	C_4_	C_5_	
concentration (wt%)	99.34	99.29	99.03	99.19	99.15	99.20	99.16	99.33	99.03	99.05	99.24	99.03	99.08	99.00	99.06	39.73

**Table 10:** Energy consumption of PCE synthesis using different methods.

	INPCE		TPCE	
Energy (kW·h)	*E*_a,INPCE_	*E*_0,INPCE_	*E*_a,TPCE_	*E*_0,TPCE_
	5.00	≈*E*_0,TPCE_	5.95	≈*E*_0,INPCE_

## Data Availability

The original contributions presented in this study are included in the article. Further inquiries can be directed to the corresponding author(s).
